# O Polimorfismo Genético do Receptor Beta-Adrenérgico Tipo 1 Ser49Gly é Preditor de Morte em Pacientes Brasileiros com Insuficiência Cardíaca

**DOI:** 10.36660/abc.20190187

**Published:** 2020-05-12

**Authors:** Felipe Neves de Albuquerque, Andrea Araujo Brandão, Dayse Aparecida Silva, Ricardo Mourilhe Rocha, Marcelo Imbroinise Bittencourt, Ana Luiza Ferreira Sales, Pedro Pimenta de Mello Spineti, Gustavo Salgado Duque, Lucas Rangel de Souza Azevedo, Roberto Pozzan, Bernardo Rangel Tura, Denilson Campos de Albuquerque

**Affiliations:** 1 Universidade do Estado do Rio de Janeiro Rio de Janeiro RJ Brasil Universidade do Estado do Rio de Janeiro, Rio de Janeiro, RJ – Brasil; 2 Hospital Samaritano Rio de Janeiro RJ Brasil Hospital Samaritano, Rio de Janeiro, RJ – Brasil; 3 Universidade do Estado do Rio de Janeiro Instituto de Biologia Rio de Janeiro RJ Brasil Universidade do Estado do Rio de Janeiro - Instituto de Biologia, Rio de Janeiro, RJ – Brasil; 4 Instituto Nacional de Cardiologia Rio de Janeiro RJ Brasil Instituto Nacional de Cardiologia – Arritmia,Rio de Janeiro, RJ – Brasil

**Keywords:** Insuficiência Cardíaca/mortalidade, Epidemiologia, Polimorfismo Genético, Receptores Adrenérgicos beta, Doenças Cardiovasculares, Hospitalização, Epinefrina/uso terapêutico, Cardiotoxicidade

## Abstract

**Fundamento:**

O papel do polimorfismo genético do receptor beta1-adrenérgico Ser49Gly (PG-Rβ1-Ser49Gly) como preditor de eventos na insuficiência cardíaca (IC) não está definido para a população brasileira.

**Objetivos:**

Avaliar a relação entre PG-Rβ1-Ser49Gly e desfechos clínicos em indivíduos com IC com fração de ejeção reduzida.

**Métodos:**

Análise secundária de prontuários de 178 pacientes e identificação das variantes do PG-Rβ1-Ser49Gly, classificadas como Ser-Ser, Ser-Gly e Gly-Gly. Avaliar sua relação com evolução clínica. Foi adotado nível de significância de 5%.

**Resultados:**

As médias da coorte foram: seguimento clínico, 6,7 anos; idade, 64,4 anos; 63,5% de homens e 55,1% brancos. A etiologia da IC foi predominantemente isquêmica (31,5%), idiopática (23,6%) e hipertensiva (15,7%). O perfil genético teve a seguinte distribuição: 122 Ser-Ser (68,5%), 52 Ser-Gly (28,7%), e 5 Gly-Gly (2,8%). Houve relação significativa entre esses genótipos e a classe funcional da New York Heart Association (NYHA) ao final do acompanhamento (p = 0,014) com o Gly-Gly associado a NYHA menos avançada. Com relação aos desfechos clínicos, houve associação significativa (p = 0,026) entre mortalidade e PG-Rβ1-Ser49Gly: o número de óbitos em pacientes com Ser-Gly (12) ou Gly-Gly (1) foi menor que com Ser-Ser (54). O alelo Gly teve um efeito protetor independente mantido após análise multivariada e foi associado à redução na chance de óbito de 63% (p = 0,03; odds ratio 0,37 – IC 0,15 a 0,91).

**Conclusão:**

A presença do PG-Rβ1 Gly-Gly associou-se a melhor evolução clínica avaliada pela classe funcional da NYHA e foi preditor de menor risco de mortalidade, independentemente de outros fatores, em seguimento de 6,7 anos. (Arq Bras Cardiol. 2020; 114(4):616-624)

## Introdução

A insuficiência cardíaca (IC) é atualmente a principal causa de internação por doenças do sistema circulatório no Sistema Único de Saúde (SUS): foram 202 mil pacientes hospitalizados em 2018 com custo de 311 milhões de reais.^1^

A estratégia atual com parâmetros clínicos, laboratoriais e de imagem para prever seu prognóstico é limitada. Sua história natural é imprevisível mesmo em pacientes fenotipicamente semelhantes.

O arsenal terapêutico é capaz de reduzir a sua mortalidade em até 60%,^2^ mas a resposta a esses fármacos é heterogênea. Foi demonstrado que a natureza genética contribui para essa variabilidade.^3^

Na fisiopatologia da IC, o papel do sistema nervoso simpático (SNS) está bem estabelecido. O receptor cardíaco beta-adrenérgico do tipo 1 (Rβ1) é a principal estrutura responsável por mediar os efeitos da epinefrina. A estimulação sustentada desse sistema determinará múltiplos efeitos deletérios,^3^ destacando-se a cardiotoxicidade.^6^

Assim, foram descritas algumas variantes genéticas que modificavam a atividade desse receptor. Um polimorfismo genético (PG) foi identificado na posição 145 do nucleotídio que resultava em substituição da serina por glicina na posição 49 do aminoácido – o PG *Ser49Gly* do Rβ1(PG-Rβ1-Ser49Gly).^7^

O PG-Rβ1-Ser49Gly foi associado a uma interferência dramática na função do Rβ1. O alelo Gly determinou maior redução no seu número ( *down-regulation* ) quando comparado ao alelo Ser.^6 , 7^ Na IC, pela exposição contínua à epinefrina, essa disfunção poderia ser clinicamente relevante. Na prática, essa mutação genética determinaria uma dessensibilização com um interessante bloqueio adrenérgico intrínseco.^8^

Assim, no contexto da IC, tivemos algumas publicações analisando o PG-Rβ1-Ser49Gly em cenários como: risco de IC,^3 , 9 - 11^ resposta a betabloqueador,^6 , 12^ desfechos ecocardiográficos,^13^ capacidade funcional,^14^ arritmia cardíaca^10 , 15^ e desfechos clínicos.^7 , 16 , 17^ Esses estudos compartilham um baixo número de pacientes e apresentam alguns achados divergentes. Em geral, o alelo Gly foi associado a uma melhor evolução clínica;^7 , 17^ no entanto, observou-se uma potencial influência da etnia sobre esses genótipos, invertendo esse comportamento benigno em algumas populações.^9^ Por essas razões, o papel desse genótipo permanece em aberto.

Portanto, é fundamental a análise do comportamento desse PG em uma população brasileira com características étnicas próprias, a fim de estabelecer o padrão desse PG para essa população aumentando a nossa (pequena) base de dados de genética atual.^10 , 16^

Este trabalho tem como objetivo avaliar a relação entre os genótipos do Ser49Gly e desfechos clínicos maiores, tais como internação por IC e óbito em indivíduos com IC com fração de ejeção reduzida.

## Métodos

### Delineamento do estudo

Estudo longitudinal de uma coorte de pacientes. Coletou-se informação do prontuário médico entre janeiro de 2015 e dezembro de 2018, desde o início do seu acompanhamento. Todos foram atendidos na mesma clínica de IC de um hospital universitário.

### População do estudo

Trata-se de uma série de casos acompanhados por 6,7 anos, sendo incluídos de forma consecutiva 178 pacientes (113 homens e 65 mulheres) com diagnóstico de IC com fração de ejeção reduzida, caracterizando-se uma amostra por conveniência.

### Critérios de inclusão

Pacientes com mais de 18 anos de idade, com IC sintomática (definida pelos Critérios de Framingham), disfunção ventricular sistólica e fração de ejeção do ventrículo esquerdo (FEVE) ≤ 50% no ecocardiograma bidimensional.

### Critérios de exclusão

Pacientes com *status* clínico desconhecido no final do trabalho.

## Método

### Análise estatística

A análise estatística é feita por meio do programa SPSS para Mac versão 25. Em todos os testes, fixou-se em 0,05 ou 5% (p < 0,05) como nível de rejeição da hipótese de nulidade e intervalo de confiança (IC) de 95%. Todas as variáveis contínuas apresentadas nas comparações tiveram distribuição normal e as medidas de tendência central e dispersão foram expressas, respectivamente, como média ± desvio padrão. Variáveis categóricas foram expressas em frequências absolutas e relativas n (%).

Foram utilizados os seguintes testes estatísticos: ANOVA One-Way, complementado pelo teste de Tukey, Chi-quadrado e Regressão Logística. Foram utilizados o teste de Levene e Kolmogorov-Smirnov para avaliar a homogeneidade das variâncias. Quando não houve homogeneidade das variâncias, foi empregado o teste Kruskal-Wallis para comparar as médias de três ou mais amostras independentes e Mann-Whitney para até duas amostras independentes.

A regressão logística binária foi utilizada para a avaliação dos desfechos clínicos estudados. Inicialmente, para a análise univariada, foram avaliadas as variáveis isoladamente com o objetivo de se identificar quais tinham relevância estatística. Posteriormente, na análise multivariada, essa avaliação foi feita de forma conjunta, como covariáveis. Foi considerado nível de significância de 95% para escolha de entrada e de 90% para remoção de variáveis no método de escolha “passo a passo".

### Etiologia da insuficiência cardíaca

As etiologias foram classificadas em cinco grupos: isquêmica, idiopática, hipertensiva, alcoólica e outras. A definição da etiologia era responsabilidade do médico assistente da clínica de IC, segundo critérios descritos previamente.^18^

### Parâmetros clínicos, laboratoriais e eletrocardiográficos

A cor da pele foi indicada pelo médico assistente e classificada em branca, preta ou parda. A classe funcional foi determinada de acordo com a NYHA, no início e no final do acompanhamento. O registro de óbito era extraído do prontuário médico e, na sua ausência, uma busca ativa era iniciada por meio de prontuário eletrônico, telefone ou bancos de dados de certidões de óbito disponíveis na internet.

### Foram considerados os exames laboratoriais mais recentes para análise estatística.

Todos os indivíduos foram submetidos a eletrocardiograma (ECG) e analisados quanto a duração do QRS, presença de bloqueio de ramo esquerdo e fibrilação atrial.

### Variáveis ecocardiográficas

Os parâmetros avaliados foram: diâmetro sistólico de VE, diâmetro diastólico de VE e fração de ejeção de VE. Foram utilizados dois exames: no início e no final do acompanhamento.

### Genotipagem

A genotipagem foi realizada por meio da técnica de reação em cadeia da polimerase e restrição da extensão do fragmento (PCR-RFLP) para o gene do Rβ1: polimorfismo 49Ser>Gly. Os detalhes desses procedimentos seguiram literatura específica.^19^

Todos os indivíduos foram testados para a presença dos alelos Ser (selvagem e mais comum) e Gly (recessivo). A partir desses alelos, eles foram classificados em Ser-Ser, Ser-Gly e Gly-Gly.

As frequências gênicas e haplotípicas foram testadas para o equilíbrio de Hardy Weinberg^20^ utilizando o *software* ARLEQUIN versão 2000.

O projeto foi aprovado pelo Comitê de Ética em Pesquisa do Hospital Universitário Pedro Ernesto em 16 de dezembro de 2009. O Termo de Consentimento Livre e Esclarecido foi assinado por todos os pacientes.

O presente estudo foi parcialmente financiado pela Fundação Carlos Chagas Filho de Amparo à Pesquisa do Estado do Rio de Janeiro (FAPERJ).

## Resultados

Características da população do estudo total e pelos PG-Rβ1

As características gerais da população estão expostas na Tabela 1 . É possível destacar: média de idade de 64,4 ± 12,8 anos (variação: 24 a 93 anos), maior prevalência de homens, cor da pele branca e de etiologia isquêmica.

**Tabela 1:** – Características da população do estudo total e pelos PG-Rβ1

Variável clínica^*^		Total	Polimorfismo genético receptor 1 Ser49Gly			

			Ser-Ser (n = 122)	Ser-Gly (n = 51)	Gly-Gly (n = 5)	p
Homens n (%)		113 (63,5)	79 (64,8)	31 (60,8)	3 (60,0)	0,873
Seguimento (anos)		6,7 ± 4,4				
Tempo de IC (anos)		8,9 ± 6,1				
Idade (anos)		64,4 ±12,8				
Cor da pele n (%)	Branca	98 (55,1)	76 (62,3)	22 (43,1)	0,0	0,003
	Preta	28 (15,7)	20 (16,4)	6 (11,8)	2 (40,0)	
	Parda	52 (29,2)	26 (21,3)	23 (45,1)	3 (60,0)	
Etiologia n (%)	DAC	56 (31,5)	43 (35,2)	12 (23,5)	1 (20,0)	0,093
	Idiopática	42 (23,6)	27 (22,1)	13 (25,5)	2 (40,0)	
	HAS	28 (15,7)	13 (10,7)	13 (25,5)	2 (40,0)	
	Álcool	19 (10,7)	12 (9,8)	7 (13,7)	0 (0,0)	
	Outras	33 (18,5)	27 (22,1)	6 (11,8)	0 (0,0)	
NYHA Inicial^†^ n (%)	I	47 (26,6)	36 (29,8)	9 (17,6)	2 (40,0)	0,334
	II	70 (39,5)	50 (41,3)	19 (37,3)	1 (20,0)	
	III	47 (26,6)	28 (23,1)	17 (33,3)	2 (40,0)	
	IV	13 (7,3)	7 (5,8)	6 (11,8)	0 (0,0)	
	Média	2,15 ± 0,9	2,05 ± 0,9	2,39 ± 0,9	2,00 ± 1,0	0,068
FEVE inicial (%)		34,8 ± 10,7	35,3 ± 11,2	33,5 ± 8,1	37,4 ± 2,1	0,54
HAS n (%)		134 (75,7)	88 (72,7)	42 (82,4)	4 (80,0)	0,395
DM n (%)		60 (33,7)	39 (32,0)	19 (37,3)	2 (40,0)	0,763
FA n (%)		41 (24,0)	29 (24,8)	12 (24,5)	0 (0,0)	0,492
Lab	Hemoglobina (mg/dL)	13,2 ± 1,9	13,2 ± 2,0	13,1 ± 1,7	13,8 ± 2,2	0,734
	Sódio (mEq/L)	139,8 ± 3,4	139,9 ± 3,4	139,8 ± 3,3	139,0 ± 4,6	0,843
	Potássio (mEq/L)	4,47 ± 0,7	4,46 ± 0,7	4,52 ± 0,6	4,38 ± 0,5	0,836
	Creatinina (mg/dL)	1,41 ± 1,0	1,50 ± 1,1	1,23 ± 0,5	1,06 ± 0,2	0,199
Tratamento	BB n (%)	173 (97,2)	118 (96,7)	50 (98,0)	5 (100,0)	0,828
	IECA n (%)	79 (44,4)	52 (42,6)	23 (45,1)	4 (80,0)	0,255
	BRA n (%)	54 (30,3)	37 (30,3)	16 (31,4)	1 (20,0)	0,87
	Espiro n (%)	83 (46,6)	52 (42,6)	27 (52,9)	4 (80,0)	0,147
	Digoxina n (%)	47 (26,4)	30 (24,6)	15 (29,4)	2 (40,0)	0,631
	Baixa adesão n (%)	81 (46,0)	52 (43,0)	27 (54,0)	2 (40,0)	0,405
	Furosemida (dose-mg)	90,8 ± 64,3	97,3 ± 66,8	81,0 ± 59,8	55,0 ± 30,0	0,22

*As variáveis numéricas estão expressas em média ± desvio-padrão; as variáveis categóricas em [n e (%)]. PG-Rβ1: polimorfismo genético receptor β1 Ser49Gly; seguimento: tempo de seguimento; tempo IC: tempo de evolução da doença desde a data do diagnóstico; DAC: doença arterial coronariana; HAS: hipertensão arterial sistêmica; NYHA: classe funcional da New York Heart Association; FEVE: fração de ejeção do ventrículo esquerdo; DM: diabetes melito; FA: fibrilação atrial; BRE: bloqueio de ramo esquerdo; Hb: hemoglobina; BB: betabloqueador; IECA: inibidor da enzima de conversão de angiotensina; BRA: bloqueador do receptor da angiotensina; Espiro: espironolactona; Lab: laboratório. †Não havia disponibilidade de dados relativos à classe NYHA inicial para 1 paciente do Grupo Ser-Ser.

O tempo de acompanhamento médio na clínica de IC foi 6,7 ± 4,4 anos.

Quanto ao perfil genético, o alelo Ser ocorreu 295 vezes (82,8%), enquanto o Gly, 61 vezes (17,2%). Com relação aos genótipos, 122 (68,5%) foram classificados como o Ser-Ser, 51 (28,7%) Ser-Gly e apenas 5 (2,8%) como Gly-Gly.

É possível destacar a diferença significativa (p = 0,003) entre os PG-Rβ1 e a cor da pele observada: houve maior prevalência de brancos entre aqueles com genotipagem Ser-Ser e praticamente um equilíbrio entre brancos e pardos com Ser-Gly como destacado na Tabela 1 .

A população estava em equilíbrio genético segundo o teorema de Hardy-Weinberg.^20^

Não houve diferenças significativas entre os genótipos para as características clínicas, da classe funcional NYHA inicial, eletrocardiográficas, ecocardiográficas, laboratoriais ou do tratamento medicamentoso conforme representado na Tabela 1 .

### Evolução clínica

Os dados sobre os desfechos clínicos estão representados na Tabela 2 .

**Tabela 2:** – Desfechos clínicos características da população do estudo total e pelos PG-Rβ1

Variável clínica^*^		Total	Polimorfismo genético receptor 1 Ser49Gly			

			Ser-Ser (n = 122)	Ser-Gly (n = 51)	Gly-Gly (n = 5)	p
NYHA Final	I	68	42	24	2	**0,014**
		38,2%	34,4%	47,1%	40,0%	
	II	57	45	9	3	
		32,0%	36,9%	17,6%	60,0%	
	III	35	19	16	0	
		19,7%	15,6%	31,4%	0,0%	
	IV	18	16	2	0	
		10,1%	13,1%	3,9%	0,0%	
	Média	2,02 ± 1,0	2,07 ± 1,0	1,92 ± 1,0	1,6 ± 0,5	0,420
FEVE Final (%)		35,4 ± 13,3	35,1 ± 13,2	35,8 ± 13,4	39,6 ± 16,2	0,751
Internação	n	74	54	18	2	0,55
	%	41,6%	44,3%	35,3%	40,0%	
Óbito	n	67	54	12	1	**0,026**
	%	37,6%	44,3%	23,5%	20,0%	
Internação + óbito	n	100	74	24	2	0,197
	%	56,2%	60,7%	47,1%	40,0%	

*As variáveis numéricas estão expressas em média ± desvio-padrão; as variáveis categóricas em [n (%)]. PG-Rβ1: polimorfismo genético receptor β1 Ser49Gly; NYHA: classe funcional da New York Heart Association; FEVE: fração de ejeção do ventrículo esquerdo.

O genótipo do Rβ1 apresentou uma relação significativa com a classe funcional da NYHA final (p = 0,014), com o Ser-Ser associado à classe funcional mais avançada. Dos dezoito pacientes em NYHA IV, o Ser-Ser foi observado em 88,9% dos casos. O PG Ser-Gly foi responsável pelos outros dois casos. Todos os cinco pacientes com genótipo Gly-Gly evoluíram com NYHA I ou II ao final do seguimento.

A média da classe funcional NYHA final foi menor que a inicial (2,15 ± 0,9 → 2,02 ± 1,0). Com relação ao caráter evolutivo, 24,9% evoluíram com melhora de classe funcional, 38,4% permaneceram estáveis e 36,7% com piora da NYHA. Não houve diferença significativa entre os PG-Rβ1 e os valores médios da NYHA ou da mudança de classe funcional durante o seguimento clínico.

### Desfechos: óbitos e internações por insuficiência cardíaca

Foram pesquisados desfechos clínicos – internação por IC e óbito – combinados e isoladamente.

O desfecho combinado “internação por IC + óbito” ocorreu em 100 pacientes (56,2%). Ele foi mais frequente no grupo Ser-Ser (60,7%) sem diferença significativa quando comparado ao Ser-Gly (47,1%) ou Gly-Gly (40,0%).

Com relação ao número de hospitalizações isoladamente, observou-se um total de 182 eventos em 74 pacientes, sem diferença significativa entre os PG-Rβ1.

**Figura 1:**
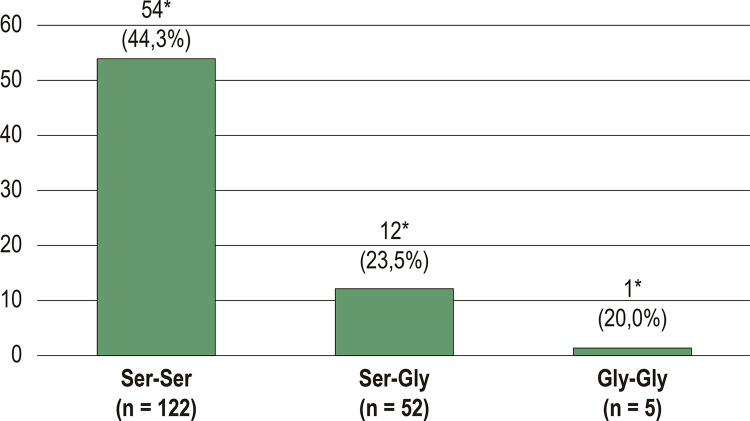
– Número de óbitos de acordo com os polimorfismos genéticos do receptor β1 Ser49Gly. Os dados foram expressos em frequências absolutas e relativas. Na comparação entre os genótipos Ser-Ser × Ser-Gly × Gly-Gly: *p = 0,026, teste qui-quadrado.

Por último, foram analisados apenas os óbitos: 67 eventos – uma taxa de mortalidade global de 37,6%. O genótipo Ser-Ser correspondeu a 80,5% desse total e apenas 1,5% dos pacientes que morreram foram genotipados com Gly-Gly. Na análise comparativa da distribuição dos óbitos pelos PG, houve uma diferença significativa (p = 0,026) entre os genótipos Ser-Ser, Ser-Gly e Gly-Gly, com taxas de mortalidade de 44,3%, 23,5% e 20,0%, respectivamente. A Tabela 2 e a Figura 1 reproduzem esses achados.

O impacto do PG-Rβ1 na mortalidade desses pacientes foi demonstrado através de análise multivariada: o alelo Gly teve um efeito protetor independente de outros fatores, após o ajuste para NYHA final, FEVE final, creatinina, baixa adesão e frequência cardíaca final. A presença de cada cópia do alelo Gly foi associada à redução na chance de óbito de 63% (p = 0,03; odds ratio 0,37 – IC 0,15 a 0,91). Esses dados estão apresentados na Tabela 3 .

**Tabela 3:** – Análise multivariada: fatores preditores de óbito

Variável	p	odds ratio
Cópia do Alelo Gly	0,030	0,37 (0,15 a 0,91)
NYHA final	0,002	2,14 (1,32 a 3,45)
FEVE final	0,002	0,94 (0,91 a 0,98)
Creatinina	0,051	1,52 (1,00 a 2,31)
Baixa adesão	0,346	1,50 (0,65 a 3,46))
FC final	0,124	1,03 (0,99 a 1,07)

*NYHA: classe funcional da New York Heart Association; FEVE: fração de ejeção de ventrículo esquerdo; FC: frequência cardíaca*

Foi possível apurar a causa *mortis* em 56% (34) dos casos: 61,8% foram relacionadas com a piora da IC; 29,4%, morte súbita; e 8,8%, outras causas. Não houve diferença entre os genótipos para a causa da morte.

## Discussão

Este trabalho descreve a relação entre os genótipos do polimorfismo genético do receptor beta1 – Ser49Gly e a evolução clínica em 178 pacientes com IC, com seguimento médio de 6,7 anos. Trata-se de trabalho com genotipagem do Ser49Gly no contexto da IC com maior tempo de seguimento já publicado. Seu principal achado foi a associação do PG-Rβ1 Gly-Gly com um efeito protetor para desfechos clínicos, com melhor evolução clínica avaliada pela classe funcional NYHA e menor risco de óbitos.

Ao fazer a comparação com outras populações brasileiras, encontramos uma distribuição alélica relativamente semelhante: o alelo Gly esteve presente em 13 a 17% dos casos de IC.^10 , 16^ Com relação aos genótipos, houve grande semelhança com o trabalho de 201 pacientes do Rio Grande do Sul,^10^ mas uma diferença com a coorte com 146 pacientes de Niterói, no Rio de Janeiro.^16^

É possível que com a intensa miscigenação da população brasileira, a cor da pele não seja um bom fator determinante do perfil genético, pois, apesar da similaridade no percentual de brancos entre o presente estudo e o de Pereira et al.,^16^ há diferença do seu perfil genético. Assim, a etnia avaliada pela cor da pele isoladamente não explicaria o alto percentual do genótipo Gly-Gly observado por esses autores. Reforçando esse ponto, estudos internacionais mostraram acentuada semelhança com a presente coorte quanto à distribuição genotípica do PG-Rβ1:^7 , 9 , 17^ 63 a 73% de indivíduos Ser-Ser, 27 a 35% Ser-Gly e 0 a 3% Gly-Gly, embora tenham sido realizados com outras etnias. O desenvolvimento de mais estudos nacionais pode ser interessante para avaliar a distribuição genotípica desse polimorfismo genético na nossa população.

Outro aspecto ainda mais relevante é a interpretação clínica desse PG. Nesse caso, foi possível demonstrar que o Gly-Gly teve associação significativa com um marcador clínico substituto: NYHA final (p=0,014). Indivíduos portadores desse genótipo tiveram melhor evolução clínica: nenhum paciente desse grupo apresentou uma classe funcional avançada no final do seguimento. Apesar de ser relativamente pequeno (cinco indivíduos), o maior tempo de seguimento em relação aos demais trabalhos permitiu a distinção de comportamentos clínicos entre os genótipos.

Como não havia diferença das características basais entre os três PG, inclusive de tratamento, a diferença da NYHA final observada nesse estudo sugere a contribuição da influência genética na fisiopatologia da cardiopatia. Assim, a genotipagem poderia indicar um subgrupo de pacientes com IC com pior evolução clínica.

Esse achado é inédito na literatura, uma vez que não há publicações em pacientes com IC relacionando o PG-R1-Ser49Gly a variáveis clínicas evolutivas como a classe funcional pela NYHA. Por isso, não é possível confrontar esse resultado com outras populações, o que seria interessante para validação desse achado.

Apesar do seu reconhecido valor prognóstico, a classe funcional da NYHA é um marcador inexato da gravidade da IC. A falta de reprodutibilidade interexaminador já foi descrita e pode limitar sua acurácia.^21^ Ela também traduz apenas um aspecto clínico da síndrome. É possível que, no futuro, seja mais interessante estudar a relação do genótipo com escores clínicos mais completos como o MAGGIC,^22^ no qual há a combinação das variáveis clínicas, laboratoriais e ecocardiográficas.

No presente estudo, a elevada taxa de mortalidade observada, 37,6%, deve-se provavelmente ao tempo de acompanhamento prolongado. Para efeito de comparação, Biolo et al.^10^ registraram 27,9% de óbitos no Rio Grande do Sul, e Pereira et al.,^16^ 12,3% de mortalidade no Rio de Janeiro. Apesar da disparidade dessas taxas, notam-se semelhanças nas características basais dessas populações: uma FEVE em torno de 30 a 35%, a maioria (65 a 75%) dos pacientes em NYHA I ou II e uma excelente terapêutica adotada. A diferença mais significativa entre os três trabalhos reside no seu tempo de acompanhamento: 80,4 meses no presente estudo, 39,8 meses^10^ e 23 meses^16^ nos estudos citados, respectivamente.

A avaliação da associação do PG-Rβ1 com óbitos mostrou que o tipo selvagem Ser-Ser concentrou a maioria desses eventos e o alelo Gly foi consistentemente associado a um efeito protetor. A presença de cada cópia do alelo Gly foi relacionada a uma redução de 63% na chance de morte. Esse efeito protetor se manteve mesmo após ajuste rigoroso para as principais variáveis utilizadas para estratificar o prognóstico em IC. Assim, em um modelo híbrido que incorporou variáveis genéticas, clínicas, laboratoriais, ecocardiográficas, de tratamento e de exame físico, o Gly-Gly permaneceu com alto valor preditivo para a menor ocorrência de óbitos.

Na revisão da literatura, os resultados são diversos, mas, em sua maioria, compatíveis com o atual. São trabalhos em que não se observou a associação PG-Rβ1-Ser49Gly e desfechos clínicos,^10 , 16 , 23^ estudos com o mesmo padrão protetor do alelo Gly^7 , 17 , 24^ e até mesmo uma publicação associando paradoxalmente o Gly a mau prognóstico na IC.^13^

Em sintonia com nossos achados, os trabalhos iniciais de Borjesson et al.^7^ (a primeira descrição desse PG-Rβ1-Ser49Gly), Forleo et al.^24^ e Magnusson et al.^17^ destacam o perfil protetor do alelo Gly: significativamente menos óbitos nos portadores dos genótipos Ser-Gly ou Gly-Gly, inclusive após ajuste para outras variáveis.

No entanto, há um trabalho indicando o oposto: o alelo Gly associado a mau prognóstico. A publicação de Wang et al.^13^ descreve o PG-Rβ1-Ser49Gly em uma população chinesa de 430 pacientes com IC e características basais similares às do presente trabalho. Os autores relacionaram o alelo Gly a piores desfechos ecocardiográficos e maior mortalidade.

O contraste entre esses resultados pode estar relacionado a um diferente impacto genético entre as etnias. Duas evidências embasam essa teoria. Primeiro, Pereira et al.^16^ identificaram, em uma população miscigenada do município de Niterói, o Ser-Ser como um fator de mau prognóstico. Esse padrão, no entanto, foi observado apenas para pacientes com cor da pele preta . Esse resultado também foi reproduzido na metanálise de Liu et al.^9^ A análise de 2.979 pacientes genotipados para os PG-R1 tipo Ar389Gly e Ser49Gly identificou um padrão específico do alelo Gly389 para cada etnia: associação a maior risco de IC em pacientes asiáticos, enquanto, em brancos, associou-se à redução desse risco.

Nessa mesma direção, o estudo A-HEFT descreveu uma melhor resposta à combinação nitrato e hidralazina para pacientes afro-americanos.^25^ Posteriormente, McNamara et al. relacionaram esse benefício a um determinado PG da óxido nítrico sintase, mais frequente em afro-americanos em comparação a brancos.^26^

Em comum, a metanálise de Liu et al.^9^ e a publicação de McNamara et al.^26^ destacam a variedade de efeitos clínicos entre etnias diferentes no contexto da IC. Isso reforça a necessidade de estudos específicos para o Brasil, uma vez que o comportamento desses PG para uma população reconhecidamente miscigenada é imprevisível.

Esses exemplos reafirmam a influência genética na história natural da IC. De maneira abrangente, reconhecemos a resposta fisiopatológica da síndrome como resultado da ativação de sistemas hormonais. No entanto, em nível molecular, receptores beta-adrenérgicos e enzimas como a óxido nítrico sintase são alguns dos fatores importantes envolvidos no remodelamento cardíaco. A alteração funcional desses e de outros agentes em decorrência de polimorfismos genéticos pode explicar essa multiplicidade de evoluções clínicas em pacientes fenotipicamente semelhantes.

O processo de resposta neuro-humoral envolve uma infinidade de elementos, cada um potencialmente sensível a mutações genéticas diversas. Assim, é provável que um painel genético englobando os principais eixos (SNS, renina-angiotensina-aldosterona e peptídio natriurético atrial) seja mais interessante que um polimorfismo específico isolado. O primeiro passo é identificar quem são os principais marcadores genéticos para cada eixo. Com relação ao SNS e o receptor beta-adrenérgico, esse trabalho reforça o papel destacado do PG Ser49Gly.

No futuro, a construção de um escore genético multissistêmico poderá se demonstrar um poderoso preditor prognóstico. Possivelmente, um escore capaz de identificar indivíduos de alto risco, mesmo no início da evolução da doença, quando muitas vezes os achados clínicos e de exames complementares ainda não estão relevantemente alterados.

O número relativamente pequeno de pacientes (178) é uma limitação do estudo e pode ter influenciado nos resultados, principalmente pelo baixo número de pacientes com genótipo Gly-Gly. No entanto, o padrão de distribuição genotípica observado foi o mesmo da maioria dos estudos, e é o estudo com o maior tempo de acompanhamento com PG-Rβ1 no contexto da IC. Destaca-se também que, mesmo com esse número reduzido, foi possível encontrar resultados com significância estatística.

Outra limitação refere-se à coleta de dados em registros de prontuários. No entanto, como todos os indivíduos são acompanhados em clínica de IC, a padronização das rotinas de atendimento e o registro das informações, bem como o atendimento realizado por médicos dedicados ao tratamento e o acompanhamento dessa síndrome, garantiram maior qualidade nas informações obtidas. Contudo, se houvesse internação em outra instituição, não havia acesso às informações, e até mesmo o número de hospitalizações pode estar subestimado. Isso pode ter determinado a ausência de diferenças estatísticas entre os genótipos e limitado a avaliação desse desfecho clínico.

## Conclusão

Em indivíduos com IC com fração de ejeção reduzida, a presença do PG-Rβ1 Gly-Gly associou-se a melhor evolução clínica avaliada pela classe funcional da NYHA e foi preditor de menor risco de mortalidade, independentemente de outros fatores, em seguimento de 6,7 anos.

Contribuição dos autores

Concepção e desenho da pesquisa: Albuquerque FN, Brandão AA, Mourilhe-Rocha R; Obtenção de dados: Albuquerque FN, Silva DA, Bittencourt MI; Análise e interpretação dos dados: Albuquerque FN, Brandão AA; Análise estatística: Albuquerque FN, Pozzan R; Obtenção de financiamento: Albuquerque FN; Redação do manuscrito: Albuquerque FN, Brandão AA, Bittencourt MI; Revisão crítica do manuscrito quanto ao conteúdo intelectual importante: Albuquerque FN, Brandão AA, Bittencourt MI, Sales ALF, Spineti PPM, Duque GS, Albuquerque D.
