# Supplementary material for: O Polimorfismo Genético do Receptor Beta-Adrenérgico Tipo 1 Ser49Gly é Preditor de Morte em Pacientes Brasileiros com Insuficiência Cardíaca
Source: Arq Bras Cardiol. 2020 May 12;114(4):616–24. [Article in Portuguese] doi: 10.36660/abc.20190187 (PMC9744341; doi:10.36660/abc.20190187)
Supplement: Translation

# Translation: Ser49Gly Beta1-Adrenergic Receptor Genetic Polymorphism as a Death Predictor in Brazilian Patients with Heart Failure

**Authors:** Felipe Neves de Albuquerque, Andrea Araujo Brandão, Dayse Aparecida Silva, Ricardo Mourilhe Rocha, Marcelo Imbroinise Bittencourt, Ana Luiza Ferreira Sales, Pedro Pimenta de Mello Spineti, Lucas Rangel de Souza Azevedo, Gustavo Salgado Duque, Roberto Pozzan, Bernardo Rangel Tura, Denilson Campos de Albuquerque

**Affiliations:** 1 Brazil Universidade do Estado do Rio de Janeiro, Rio de Janeiro, RJ – Brazil; 2 Brazil Hospital Samaritano, Rio de Janeiro, RJ – Brazil; 3 Brazil Universidade do Estado do Rio de Janeiro - Instituto de Biologia, Rio de Janeiro, RJ – Brazil; 4 Brazil Instituto Nacional de Cardiologia – Arritmia, Rio de Janeiro, RJ – Brazil

**Keywords:** Heart Failure/mortality, Epidemiology, Polymorfism, Geetic, Receptors,Adreneic, beta, cardiovascular Dieases, Hospitalization, Epinephrine/therapeutic use, Cardiotoxicity

## Abstract

**Background:**

The role of Ser49Gly beta1-adrenergic receptor genetic polymorphism (ADBR1-GP-Ser49Gly) as a predictor of death in heart failure (HF) is not established for the Brazilian population.

**Objectives:**

To evaluate the association between ADBR1-GP-Ser49Gly and clinical outcomes in individuals with HF with reduced ejection fraction.

**Methods:**

Secondary analysis of medical records of 178 patients and genotypes of GPRβ1-Ser49Gly variants, classified as Ser-Ser, Ser-Gly and Gly-Gly. To evaluate their association with clinical outcome. A significance level of 5% was adopted.

**Results:**

Cohort means were: clinical follow-up 6.7 years, age 63.5 years, 64.6% of men and 55.1% of whites. HF etiologies were predominantly ischemic (31.5%), idiopathic (23.6%) and hypertensive (15.7%). The genetic profile was distributed as follows: 122 Ser-Ser (68.5%), 52 Ser-Gly (28.7%) and 5 Gly-Gly (2.8%). There was a significant association between these genotypes and mean NYHA functional class at the end of follow-up (p = 0.014) with Gly-Gly being associated with less advanced NYHA. In relation to the clinical outcomes, there was a significant association (p = 0.026) between mortality and GPRβ1-Ser49Gly: the number of deaths in patients with Ser-Gly (12) or Gly-Gly (1) was lower than in those with Ser-Ser (54). The Gly allele had an independent protective effect maintained after multivariate analysis and was associated with a reduction of 63% in the risk of death (p = 0.03; Odds Ratio 0.37 – CI 0.15–0.91).

**Conclusion:**

The presence of β1-AR-GP Gly-Gly was associated with better clinical outcome evaluated by NYHA functional class and was a predictor of lower risk of mortality, regardless of other factors, in a 6.7-year of follow-up. (Arq Bras Cardiol. 2020; 114(4):613-615)

## Introduction

Heart failure (HF) is currently the main cause of hospital admissions due to circulatory diseases in the Brazilian public healthcare system: 202,000 patients were admitted in 2018, costing BRL 311 million.^1^

The current strategy with clinical, laboratory and imaging parameters to predict prognosis is limited. The natural history of HF is unpredictable, even in phenotypically similar patients.

The therapies available are capable of reducing mortality by up to 60%,^2^ but response to these medical treatments is heterogeneous. It has been demonstrated that genetic nature influences this variability.^3 - 5^

In the pathophysiology of HF, the role of the Sympathetic Nervous System (SNS) is well established. Cardiac beta1-adrenergic receptor (Rβ1) is the main structure responsible for mediating the effects of adrenaline. Sustained stimulation of this system determines multiple deleterious effects,^3^ especially cardiotoxicity.^6^

Accordingly, some genetic variants that modified the activity of this receptor have been described. A genetic polymorphism (GP) was identified at position 145 of the nucleotide that resulted in the substitution of Serine for Glycine at position 49 of the amino acid – GPRβ1-Ser49Gly.^7^

GPRβ1-Ser49Gly was associated with a dramatic interference with Rβ1 function. The Gly allele determined greater reduction in its number (down-regulation) compared with the Ser allele.^6 , 7^ Because of the continuous exposure to adrenaline, this dysfunction could be clinically relevant in HF. In practice, this genetic mutation would determine desensitization with a relevant intrinsic adrenergic block.^8^

Accordingly, in the context of HF, some publications analyzed GPRβ1-Ser49Gly in scenarios that included: risk of HF,^3 , 9 - 11^ beta-blocker response,^6 , 12^ echocardiographic outcomes,^13^ functional capacity,^14^ cardiac arrhythmia^10 , 15^ and clinical outcomes.^7 , 16 , 17^ These studies include a small number of patients and present some inconsistent findings. In general, the Gly allele was associated with better clinical outcome;^7 , 17^ however, a potential influence of ethnicity on these genotypes was observed, inverting this benign behavior in some populations.^9^ For these reasons, the role of this genotype is still unknown.

Therefore, it is of paramount importance to analyze the behavior of this GP in a Brazilian population with its own ethnic characteristics, in order to establish the pattern of this GP for our population, increasing our (small) current genetic database.^10 , 16^

The objective of this study is to evaluate the association between the Ser49Gly genotypes and major clinical outcomes, such as hospital admissions due to HF and death in individuals with HF with reduced ejection fraction.

## Methods

### Study design

Longitudinal study of a cohort of patients. Information was collected from medical records dated between January 2015 and April 2018, since the beginning of follow-up. All patients were seen at the same HF clinic of a university hospital.

### Study population

This is a series of cases followed for 6.7 years, which consecutively included 178 patients (113 men and 65 women) diagnosed with HF with reduced ejection fraction, being characterized as a convenience sampling.

### Inclusion criteria

Patients aged 18 or older, with symptomatic HF (defined by the Framingham criteria), systolic ventricular dysfunction and left ventricular ejection fraction (LVEF) ≤ 50% on two-dimensional echocardiography.

### Exclusion criteria

Patients with unknown clinical status at the end of the study.

## Method

### Statistical analysis

Statistical analysis was performed using SPSS for Mac, version 25. For all of the tests, 0.05 or 5% (p < 0.05) was defined as the rejection level of the null hypothesis and 95% confidence interval (CI). The measures of central tendency were expressed as mean ± standard deviation. Categorical variables were expressed in absolute and relative frequencies n(%).

The following statistical tests were used: One-way ANOVA complemented by Tukey’s test, chi-square test and logistic regression. To evaluate the homogeneity of variances, Levene’s test was used. When there was no homogeneity of variances, the Kruskal-Wallis test was used to compare the means of three or more independent samples and Mann-Whitney test was used for up to two independent samples.

Binary logistic regression was used to evaluate the clinical outcomes studied. Initially, the variables were evaluated separately in order to identify which ones were statistically relevant. Subsequently, they were evaluated together as covariables. A 95% significance level was considered for entry in the model and 90% for the removal of variables in the stepwise method of choice.

### Heart failure etiology

The etiologies were classified into five groups: ischemic, idiopathic, hypertensive, alcoholic and others. The attending physician at the HF service was in charge of defining the etiology according to previously established criteria.^18^

### Clinical, laboratory and echocardiographic parameters

Skin color was determined by the attending physician and classified as white, black or other. Functional class was determined according to the New York Heart Association (NYHA) at the beginning and at the end of follow-up. Death registries were taken from the medical records and, if no records were available, an active search was conducted on the electronic medical records, by phone call or on death certificate databases available on the Internet.

### The most recent laboratory tests were considered for statistical analysis.

All individuals had their electrocardiograms (ECG) analyzed for QRS duration, presence of left bundle branch block and atrial fibrillation.

### Echocardiographic variables

The parameters evaluated were: LV systolic diameter, LV diastolic diameter and LV ejection fraction. Two tests were used: one at the beginning and another at the end of follow-up.

### Genotyping

Genotyping was performed using polymerase chain reaction and restriction fragment length polymorphism (PCR-RFLP) for the Rβ1 gene: 49Ser>Gly polymorphism. The details of these procedures followed specific literature.^19^

All individuals were tested for the presence of Ser (wild and most common) and Gly (recessive) alleles. Based on the presence of these alleles, the individuals were classified into Ser-Ser, Ser-Gly and Gly-Gly.

Gene and haplotype frequencies were tested for the Hardy-Weinberg equilibrium,^20^ using the software ARLEQUIN version 2000.

The project was approved by the Research Ethics Committee of Hospital Universitário Pedro Ernesto on 12/16/2009. An Informed Consent Form (ICF) was signed by all patients.

This study was partially funded by FAPERJ (Fundação Carlos Chagas Filho de Amparo à Pesquisa do Estado do Rio de Janeiro).

## Results

### Characteristics of the sample population and GP-Rβ1

Table 1 shows the general characteristics of the study population. Mean age of 64.4 ± 12.8 years (variation: 24–93 years), a higher prevalence of males, white skin color and ischemic etiology were observed.

**Table 1:** — Baseline characteristics of the population according to the genetic polymorphisms of Ser49Gly β1-adrenergic receptor

Clinical Variable*		Total	Ser49Gly β1 Genetic Polymorphism			

			Ser-Ser (n = 122)	Ser-Gly (n = 51)	Gly-Gly (n = 5)	p
Men n %		113 (63.5%)	79 (64.8%)	31 (60.8%)	3 (60.0%)	0.873
Follow-up (years)		6.7 ± 4.4				
Duration of HF (months)		8.9 ± 6.1				
Age (years)		64.4 ± 12.8				
Skin Color	White	98 (55.1%)	76 (62.3%)	22 (43.1%)	0 (0.0%)	0.003
	Black	28 (15.7%)	20 (16.4%)	6 (11.8%)	2 (40.0%)	
	Other	52 (29.2%)	26 (24.3%)	23 (45.1%)	3 (60.0%)	
Etiology	CAD	56 (31.5%)	43 (35.2%)	12 (23.5%)	1 (20.0%)	0.093
	Idiopathic	42 (23.6%)	27 (22.1%)	13 (25.5%)	2 (40.0%)	
	Hypert	28 (15.7%)	13 (10.7%)	13 (25.5%)	2 (40.0%)	
	Alcohol	19 (10.7%)	12 (9.8%)	7 (13.7%)	0 (0.0%)	
	Other	33 (18.5%)	27 (22.1%)	6 (11.8%)	0 (0.0%)	
Baseline NYHA^†^	I	47 (26.6%)	36 (29.8%)	9 (17.6%)	2 (40.0%)	0.334
	II	70 (39.5%)	50 (41.3%)	19 (37.3%)	1 (20.0%)	
	III	47 (26.6%)	28 (23.1%)	17 (33.3%)	2 (40.0%)	
	IV	13 (7.3%)	7 (5.8%)	6 (11.8%)	0 (0.0%)	
	Mean	2.15 ± 0.9	2.05 ± 0.9	2.39 ± 0.9	2.0 ± 1.0	0.068
Baseline LVEF (%)		34.8 ± 10.7	35.3 ± 11.2	33.5 ± 8.1	37.4 ± 2.1	0.54
Hypert	n %	134 (75.7%)	88 (72.7%)	42 (82.4%)	4 (80.0%)	0.395
DM	n %	60 (33.7%)	39 (32.0%)	19 (37.3%)	2 (40.0%)	0.763
AF	n %	41 (24.0%)	29 (24.8%)	12 (24.5%)	0 (0.0%)	0.492
Lab	Hemoglobin (mg/dL)	13.2 ± 1.9	13.2 ± 2.0	13.1 ± 1.7	13.8 ± 2.2	0.734
	Sodium (mEq/L)	139.8 ± 3.4	139.9 ± 3.4	139.8 ± 3.3	139.0 ± 4.6	0.843
	Potassium (mEq/L)	4.47 ± 0.7	4.46 ± 0.7	4.52 ± 0.6	4.38 ± 0.5	0.836
	Creatinine (mg/dL)	1.41 ± 1.0	1.50 ± 1.1	1.23 ± 0.5	1.06 ± 0.2	0.199
Treatment	BB n %	173 (97.2%)	118 (96.7%)	50 (98.0%)	5 (100.0%)	0.828
	ACEI n %	79 (44.4%)	52 (42.6%)	23 (45.1%)	4 (80.0%)	0.255
	ARB n %	54 (30.3%)	37 (30.3%)	16 (31.4%)	1 (20.0%)	0.87
	Spiro n %	83 (46.6%)	52 (42.6%)	27 (52.9%)	4 (80.0%)	0.147
	Digox n %	47 (26.4%)	30 (24.6%)	15 (29.4%)	2 (40.0%)	0.631
	Low adherence n %	81 (46.0%)	52 (43.0%)	27 (54.0%)	2 (40.0%)	0.405
	Furosemide (dose-mg)	90.8 ± 64.3	97.3 ± 66.8	81.0 ± 59.8	55.0 ± 30.0	0.22

*Numerical variables are expressed as mean ± standard deviation; categorical variables expressed as [n e (%)]. Follow-up: follow-up time (in years); duration of HF: duration of disease course since the date of diagnosis (in years); CAD: Coronary Artery Disease; Hypert: Systemic Arterial Hypertension; NYHA: New York Heart Association Functional Class; LVEF: Left Ventricular Ejection Fraction; DM: Diabetes Mellitus; AF: Atrial Fibrillation; LBBB: Left Bundle Branch Block; Hb: Hemoglobin (in mg/dL); BB: Betablocker; ACEI: Angiotensin-converting enzyme inhibitor; ARB: Angiotensin receptor blocker; Spiro: Spironolactone; Digox: Digoxin.^†^Baseline NYHA class data were not available for 1 patient from the Ser-Ser group.

The mean follow-up time at the HF clinic was 6.7 ± 4.4 years.

As for the genetic profile, the Ser allele occurred 295 times (82.8%) whereas the Gly allele occurred 61 times (17.2%). In relation to the genotypes, 122 (68.5%) were classified as Ser-Ser, 51 (28.7%) as Ser-Gly and only 5 (2.8%) as Gly-Gly.

A significant difference (p = 0.003) was found between GP-Rβ1 and skin color: there was a higher prevalence of whites among those with Ser-Ser genotype and virtually an equilibrium between Ser-Gly individuals with white skin and others, as shown in Table 1 .

The population was in genetic equilibrium, according to the Hardy-Weinberg theorem.^20^

There were no significant differences between the genotypes regarding clinical characteristics, baseline NYHA functional class, electrocardiographic, echocardiographic and laboratory characteristics or medical treatment, as shown in Table 1 .

### Clinical outcome

Clinical outcome data are shown in Table 2 .

**Table 2:** – Clinical outcomes according to the genetic polymorphisms of Ser49Gly β1-adrenergic receptor

Clinical Variable^*^		Total	Ser49Gly β1 Genetic Polymorphism			

			Ser-Ser (n = 122)	Ser-Gly (n = 51)	Gly-Gly (n = 5)	p
Final NYHA	I	68	42	24	2	**0.014**
		38.2%	34.4%	47.1%	40.0%	
	II	57	45	9	3	
		32.0%	36.9%	17.6%	60.0%	
	III	35	19	16	0	
		19.7%	15.6%	31.4%	0.0%	
	IV	18	16	2	0	
		10.1%	13.1%	3.9%	0.0%	
	Mean	2.02 ± 1.0	2.07 ± 1.0	1.92 ± 1.0	1.6 ± 0.5	0.420
Final LVEF (%)		35.4 ± 13.3	35.1 ± 13.2	35.8 ± 13.4	39.6 ± 16.2	0.751
Hospital admission	n	74	54	18	2	0.55
	%	41.6%	44.3%	35.3%	40.0%	
Death	n	67	54	12	1	**0.026**
	%	37.6%	44.3%	23.5%	20.0%	
Hospital admission + Death	n	100	74	24	2	0.197
	%	56.2%	60.7%	47.1%	40.0%	

*Numerical variables are expressed as mean ± standard deviation; categorical variables expressed as [n e (%)]. NYHA: New York Heart Association Functional Class;

The Rβ1 genotype showed a significant association with the final NYHA functional class (p = 0.014), with Ser-Ser being associated with the most advanced functional class. Of the eighteen NYHA IV patients, Ser-Ser was found in 88.9% of the cases. The Ser-Gly GP was responsible for the other two cases. All five patients with Gly-Gly genotype progressed with NYHA I or II at the end of the follow-up.

Mean NYHA functional class was lower than baseline (2.15 ± 0.9 → 2.02 ± 1.0). As for outcomes, 24.9% showed improved functional class, 38.4% remained stable and 36.7% showed NYHA worsening. There was no significant difference between GP-Rβ1 and NYHA mean values or change in functional class during clinical follow-up.

### Outcomes: Deaths and hospital admissions due to HF

The clinical outcomes of hospital admission due to HF and death were investigated both separately and in association.

Combined outcome of admission due to HF+Death occurred in 100 patients (56.2%). It was more frequent in the Ser-Ser group (60.7%) with no significant difference when comparing Ser-Gly (47.1%) with Gly-Gly (40.0%) cases.

In relation to the number of hospitalizations alone, 182 events were observed in 74 patients, with no significant difference between GP-Rβ1 types.

Finally, deaths only were analyzed: 67 events – an overall mortality rate of 37.6%. The Ser-Ser genotype accounted for 80.5% of these deaths and only 1.5% of patients who died had the Gly-Gly genotype. In the comparative analysis of the distribution of deaths by GP, there was a significant difference (p = 0.026) between the genotypes Ser-Ser, Ser-Gly and Gly-Gly, with mortality rates of 44.3%, 23.5% and 20.0%, respectively. Table 2 and Figure 1 depict these findings.

**Figure 1:** – Distribution of number of deaths according to Ser49Glyβ1 genetic polymorphism. Data were expressed in absolute and relative frequencies. In the comparison of Ser-Ser x Ser-Gly x Gly-Gly genotypes: *p = 0.026, chi-square test.

The impact of GP-Rβ1 on the mortality of these patients was shown through multivariate analysis: the Gly allele had a protective effect independent of other factors after adjustment for final NYHA, final LVEF, creatinine, low adherence and final heart rate. The presence of each copy of the Gly allele was associated with risk of death reduced by 63% (p = 0.03; Odds Ratio 0.37 – CI 0.15–0.91). These data are shown in Table 3 .

**Table 3:** – Multivariate Analysis: Predictors of death

Variable	p	Odds ratio
Copy of Gly allele	0.030	0.37 (0.15–0.91)
Final NYHA	0.002	2.14 (1.32–3.45)
Final LVEF	0.002	0.94 (0.91–0.98)
Creatinine	0.051	1.52 (1.00–2.31)
Low adherence	0.346	1.50 (0.65–3.46))
Final HR	0.124	1.03 (0.99–1.07)

*NYHA: New York Heart Association Functional Class; LVEF: left ventricular ejection fraction; HR: heart rate*

The cause of death was determined in 56% (34) of the cases: 61.8% were related to HF worsening, 29.4% of sudden deaths and 8.8% due to other causes. There was no difference between the genotypes regarding the cause of death.

## Discussion

This study describes the association between the Beta-1 Genetic Polymorphism Ser49Gly genotypes and clinical outcome in 178 patients with HF, with mean follow-up of 6.7 years. Of all the studies published on Ser49Gly genotyping in the context of HF, this one has the longest follow-up time. Its main finding was the association of Gly-Gly GP-Rβ1 with a protective effect for clinical outcomes, with better clinical outcome evaluated by NYHA functional class and lower risk of death.

When we compared with other Brazilian populations, we found a relatively similar allelic distribution: the Gly allele was present in 13 to 17% of the HF cases.^10 , 16^ In relation to the genotypes, it was largely similar to a study including 201 patients from the state of Rio Grande do Sul^10^ but different from the cohort of 146 patients from the municipality of Niterói, in the state of Rio de Janeiro.^16^

Due to the intense miscegenation of the Brazilian population, skin color is probably not a good determinant of the genetic profile, as despite the similarity in the percentage of whites between this study and that of Pereira et al.,^16^ there is a difference in their genetic profile. Thus, ethnicity assessed by skin color alone could not explain the high percentage of the Gly-Gly genotype found by Pereira et al.^16^ Stressing this point, international studies have shown a strong similarity with this cohort, as to the genotypic distribution of GP-Rβ1:^7 , 9 , 17^ 63 to 73% of Ser-Ser, 27 to 35% of Ser-Gly and 0 to 3% of Gly-Gly individuals, although the studies included other ethnicities. It may be worth conducting other national studies in order to evaluate the genotypic distribution of this genetic polymorphism in our population.

Another even more relevant aspect is the clinical interpretation of this GP. In this case, it was possible to demonstrate that Gly-Gly had a significant association with a surrogate clinical marker: final NYHA (p = 0.014). Individuals with this genotype had a better clinical outcome: no patient in this group showed advanced functional class at the end of the follow-up. Although it is a relatively small number of patients (five individuals), the longer follow-up time compared with other studies allowed the distinction of clinical behaviors among the genotypes.

Considering that there were no baseline clinical differences among the three GPs, including treatment, the difference in the final NYHA found in this study suggests that genetic variations could influence the pathophysiology of heart disease. Therefore, genotyping could identify a subgroup of patients with HF with worse clinical outcome.

This finding is an original one in the literature, as there are no publications on patients with HF correlating GPRβ1-Ser49Gly to clinical outcomes, such as NYHA functional class. Because of that, it is not possible to compare this result with other populations, which would be appropriate to validate this finding.

Despite its recognized prognostic value, NYHA functional class is an inaccurate marker of HF severity. The lack of inter-examiner reproducibility has been described and may limit its accuracy.^21^ It also translates only one clinical aspect of the syndrome. In the future, it may be more appropriate to study the association of the genotype with more complete clinical scores, such as MAGGIC,^22^ in which there is a combination of clinical, laboratory and echocardiographic variables.

The high mortality rate found in this study – 37.6% – is probably due to the long follow-up time. For comparison purposes, Biolo et al.^10^ found a mortality rate of 27.9% in Rio Grande do Sul and Pereira et al.^16^ found 12.3% in Rio de Janeiro. Despite the disparity between these rates, there are similarities in the baseline characteristics of these populations: LVEF of approximately 30–35%, the majority (65–75%) of patients in NYHA I or II and an optimized therapy adopted. The most significant difference between the three studies is their follow-up time: 80.4 months in this study, and 39.8 months^10^ and 23 months^16^ in the abovementioned studies, respectively.

Assessment of the association of GP-Rβ1 with mortality showed that the wild Ser-Ser allele concentrated most of these events and the Gly allele was consistently associated with a protective effect. The presence of each copy of the Gly allele was associated with a 63% reduction in the risk of death. This protective effect was maintained even after strict adjustment for the main variables used to stratify HF prognosis. Accordingly, in a hybrid model that incorporated genetic, clinical, laboratory, echocardiographic, treatment and physical examination variables, Gly-Gly remained with a high predictive value for the lower occurrence of deaths.

In the literature review, the results are diverse, but mostly consistent with the current one. Those include studies that have found no association between GPRβ1-Ser49Gly and clinical outcomes,^10 , 16 , 23^ studies with the same protective pattern as the Gly allele^7 , 17 , 24^ and even a paper paradoxically associating Gly with poor prognosis in HF.^13^

In line with our findings, the first studies of Borjesson et al.^7^ (the first description of this GPRβ1-Ser49Gly), Forleo et al.^24^ and Magnusson et al.^17^ describe the protective profile of the Gly allele: significantly fewer deaths were observed with the genotypes Ser-Gly or Gly-Gly, even after adjusting for other variables.

However, there is a study describing the opposite, i.e., the Gly allele associated with poor prognosis. Wang et al.^13^ described GPR1-Ser49Gly in a Chinese population of 430 patients with HF and baseline characteristics similar to those of this study. The authors associated the Gly allele to worse echocardiographic outcomes and higher mortality.

The contrast between these findings may be related to a different genetic impact on the ethnicities. Two pieces of evidence underlie this theory. Firstly, Pereira et al.^16^ identified Ser-Ser as a factor of poor prognosis in a multi-ethnic population from the city of Niterói, state of Rio de Janeiro. Nevertheless, this pattern was only found in patients with black skin. This finding was also reproduced in the meta-analysis of Liu et al.^9^ The analysis of 2,979 patients genotyped for Ar389Gly and Ser49Gly GP-Rβ1 identified a specific pattern of the Gly389 allele for each ethnicity: association with higher risk of HF in Asian patients, while in whites, it was associated with a reduction of this risk.

Along the same lines, the A-HEFT study described better response to nitrate and hydralazine combination for African-American patients.^25^ Subsequently, McNamara et al. associated this benefit to a particular GP of Nitric Oxide Synthase, more frequent in African Americans compared to whites.^26^

Likewise, the meta-analysis of Liu et al.^9^ and the study by McNamara et al.^26^ describe the variety of clinical effects among different ethnicities in the context of HF. This reinforces the need for specific studies targeted at Brazilian patients, as the behavior of these GPs for a population that is recognized as miscegenated is unpredictable.

These examples reaffirm the genetic influence on the natural history of HF. Generally speaking, we acknowledge the pathophysiological response of the syndrome as a result of the activation of hormonal systems. However, at the molecular level, beta-adrenergic receptors and enzymes, such as nitric oxide synthase, are some of the important factors implicated in cardiac remodeling. The functional modification of these and other agents due to genetic polymorphisms may explain these multiple clinical outcomes in phenotypically similar patients.

The process of neurohumoral response involves a multitude of elements, each potentially sensitive to diverse genetic mutations. In view of that, a genetic panel including the main systems (sympathetic nervous system, renin-angiotensin-aldosterone system and atrial natriuretic peptide) is likely to be more appropriate than a specific isolated polymorphism. The first step is to identify the main genetic markers for each system. In relation to the sympathetic nervous system and the beta-adrenergic receptor, this study stresses the prominent role of the Ser49Gly GP.

In the future, the construction of a multisystemic genetic score may prove to be a powerful prognostic predictor. Possibly, a score capable of identifying high-risk individuals, even at the onset of disease, when clinical findings and complementary tests are not yet significantly abnormal.

The relatively small number of patients (178) is a limitation in this study and may have influenced the results, especially due to the low number of patients with the Gly-Gly genotype. However, the genotypic distribution pattern observed was the same in most studies and this is the study with the longest follow-up time with GP-Rβ1 in the context of HF. It is also worth noting that, despite this reduced number, it was possible to find results with statistical significance.

Another limitation refers to the collection of data from medical records. However, since all individuals are followed at a HF clinic, standardization of care routines and information records, as well as the care provided by the doctors involved with the treatment and follow-up of this syndrome, ensured higher quality of the information obtained. Nevertheless, if hospitalization occurred in another institution, there was no access to information and even the number of hospitalizations may be underestimated. This may have determined the absence of statistical differences between the genotypes and limited the evaluation of this clinical outcome.

## Conclusions

In patients with HF with reduced ejection fraction, the presence of Gly-Gly GP-Rβ1 was associated with better clinical outcome assessed by NYHA functional class and was a predictor of lower risk of mortality, regardless of other factors in 6.7 years of follow-up.
